# Administration of Exogenous Melatonin Improves the Diurnal Rhythms of the Gut Microbiota in Mice Fed a High-Fat Diet

**DOI:** 10.1128/mSystems.00002-20

**Published:** 2020-05-19

**Authors:** Jie Yin, Yuying Li, Hui Han, Jie Ma, Gang Liu, Xin Wu, Xingguo Huang, Rejun Fang, Kenkichi Baba, Peng Bin, Guoqiang Zhu, Wenkai Ren, Bie Tan, Gianluca Tosini, Xi He, Tiejun Li, Yulong Yin

**Affiliations:** a College of Animal Science and Technology, Hunan Co-Innovation Center of Animal Production Safety, Hunan Agricultural University, Changsha, China; b Key Laboratory of Agro-ecological Processes in Subtropical Region, Institute of Subtropical Agriculture, Hunan Provincial Key Laboratory of Animal Nutritional Physiology and Metabolic Process, Chinese Academy of Sciences, Changsha, Hunan, China; c Department of Pharmacology and Toxicology, Neuroscience Institute, Morehouse School of Medicine, Atlanta, Georgia, USA; d College of Veterinary Medicine, Yangzhou University, Yangzhou, China; e Guangdong Provincial Key Laboratory of Animal Nutrition Control, Institute of Subtropical Animal Nutrition and Feed, College of Animal Science, South China Agricultural University, Guangzhou, China; Luxembourg Centre for Systems Biomedicine

**Keywords:** melatonin, circadian clock, gut microbiota, lipid dysmetabolism

## Abstract

Melatonin, a circadian hormone, has been reported to improve host lipid metabolism by reprogramming the gut microbiota, which also exhibits rhythmicity in a light/dark cycle. However, the effect of the administration of exogenous melatonin on the diurnal variation in the gut microbiota in mice fed a high-fat diet (HFD) is unclear. Here, we further confirmed the antiobesogenic effect of melatonin on mice fed an HFD for 2 weeks. Samples were collected every 4 h within a 24-h period, and diurnal rhythms of clock gene expression (*Clock*, *Cry1*, *Cry2*, *Per1*, and *Per2*) and serum lipid indexes varied with diurnal time. Notably, *Clock* and triglycerides (TG) showed a marked rhythm in the control in melatonin-treated mice but not in the HFD-fed mice. The rhythmicity of these parameters was similar between the control and melatonin-treated HFD-fed mice compared with that in the HFD group, indicating an improvement caused by melatonin in the diurnal clock of host metabolism in HFD-fed mice. Moreover, 16S rRNA gene sequencing showed that most microbes exhibited daily rhythmicity, and the trends were different for different groups and at different time points. We also identified several specific microbes that correlated with the circadian clock genes and serum lipid indexes, which might indicate the potential mechanism of action of melatonin in HFD-fed mice. In addition, effects of melatonin exposure during daytime or nighttime were compared, but a nonsignificant difference was noticed in response to HFD-induced lipid dysmetabolism. Interestingly, the responses of microbiota-transplanted mice to HFD feeding also varied at different transplantation times (8:00 and 16:00) and with different microbiota donors. In summary, the daily oscillations in the expression of circadian clock genes, serum lipid indexes, and the gut microbiota appeared to be driven by short-term feeding of an HFD, while administration of exogenous melatonin improved the composition and diurnal rhythmicity of some specific gut microbiota in HFD-fed mice.

**IMPORTANCE** The gut microbiota is strongly shaped by a high-fat diet, and obese humans and animals are characterized by low gut microbial diversity and impaired gut microbiota compositions. Comprehensive data on mammalian gut metagenomes shows gut microbiota exhibit circadian rhythms, which is disturbed by a high-fat diet. On the other hand, melatonin is a natural and ubiquitous molecule showing multiple mechanisms of regulating the circadian clock and lipid metabolism, while the role of melatonin in the regulation of the diurnal patterns of gut microbial structure and function in obese animals is not yet known. This study delineates an intricate picture of melatonin-gut microbiota circadian rhythms and may provide insight for obesity intervention.

## Abstract (precis)

The gut microbiota is strongly shaped by a high-fat diet, and obese humans and animals are characterized by low gut microbial diversity and impaired gut microbiota compositions. Comprehensive data on mammalian gut metagenomes shows gut microbiota exhibit circadian rhythms, which is disturbed by a high-fat diet. On the other hand, melatonin is a natural and ubiquitous molecule showing multiple mechanisms of regulating the circadian clock and lipid metabolism, while the role of melatonin in the regulation of the diurnal patterns of gut microbial structure and function in obese animals is not yet known. This study delineates an intricate picture of melatonin-gut microbiota circadian rhythms and may provide insight for obesity intervention.

## INTRODUCTION

Melatonin is a natural hormone that is mainly secreted by the pineal gland, where its synthesis is driven by the master circadian clock located in the suprachiasmatic nucleus of the hypothalamus (1, 2). Melatonin synthesis is activated by darkness and inhibited by light; thus, this hormone is a key regulator of the circadian network (3–7). In addition, melatonin is also involved in various physiological processes (i.e., antioxidant activity, bone formation, reproduction, cardiovascular function, and immune regulation) and has been confirmed to have therapeutic effects on gastrointestinal diseases, psychiatric disorders, cardiovascular diseases, and cancers (8–10). More recently, a few studies have reported that melatonin receptor 1 knockout mice show insulin and leptin resistance (11, 12), indicating a role of melatonin and its downstream signals in energy metabolism. Additionally, melatonin injection in lipopolysaccharide-induced endotoxemia markedly improves energy metabolism by enhancing ATP production (13). A similar effect of melatonin is also observed in diabetes, where lower melatonin secretion is independently associated with a higher risk of developing type 2 diabetes (14, 15). These findings indicate an interaction between melatonin signaling and metabolic diseases. Indeed, Xu et al. also identified the antiobesity effect of melatonin on high-fat diet (HFD)-induced obesity in a murine model, reporting improvement in liver steatosis, low-grade inflammation, insulin resistance, and gut microbiota diversity and composition (16). We further confirmed the underlying mechanism of action of melatonin in HFD-induced lipid dysmetabolism, which may be associated with reprogramming of gut microbial functions, especially *Bacteroides*- and *Alistipes*-mediated acetic acid production (17).

The gut microbiota is strongly shaped by HFDs, and obese humans and animals are characterized by low gut microbial diversity and impaired gut microbiota compositions, especially in terms of *Firmicutes* and *Bacteroidetes* abundances (18–25). Interestingly, several reports have revealed that the gut microbiota and its metabolites exhibit circadian rhythms, which are driven by HFDs (26–30). Additionally, some microbes have been reported to be sensitive to melatonin (31), but the role of melatonin in the regulation of the diurnal patterns of gut microbial structure and function and whether gut microbiota oscillations are associated with the antiobesity effect of melatonin are not yet known.

In this study, we further analyzed the short-term effect of HFD feeding on diurnal variations in the gut microbiota and the relationship between gut microbiota oscillations and the expression of circadian clock genes and serum lipids.

## RESULTS

### Melatonin alleviates adipose accumulation in HFD-fed mice.

**FIG 1:**
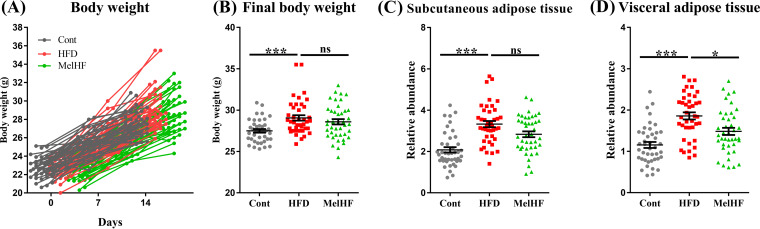
Effect of melatonin treatment on body weight and lipid accumulation in HFD-fed mice. Body weights (A), final body weights (B), relative weights of subcutaneous adipose tissues compared to body weights (C), and relative weights of visceral adipose tissues compared to body weights (D) (*n* = 42). Values are presented as the means ± SEMs. Differences were assessed by Bonferroni’s test and denoted as follows: *, *P* < 0.05; ***, *P* < 0.001; ns, *P* > 0.05.

Body weights were recorded in the present study, and the results showed an increase in final body weight after 2 weeks of HFD feeding (*P* < 0.001) (Fig. 1A and B). Our previous study confirmed that administration of exogenous melatonin improved subcutaneous adipose accumulation in HFD-fed mice (17), and the relative weight of subcutaneous adipose (*P* > 0.05) tended to be low in the HFD plus melatonin (MelHF) group. The amount of visceral adipose tissue (*P* < 0.05) was markedly reduced in the MelHF group in this study (Fig. 1C and D).

### Melatonin affects clock gene expression in HFD-fed mice.

**FIG 2:**
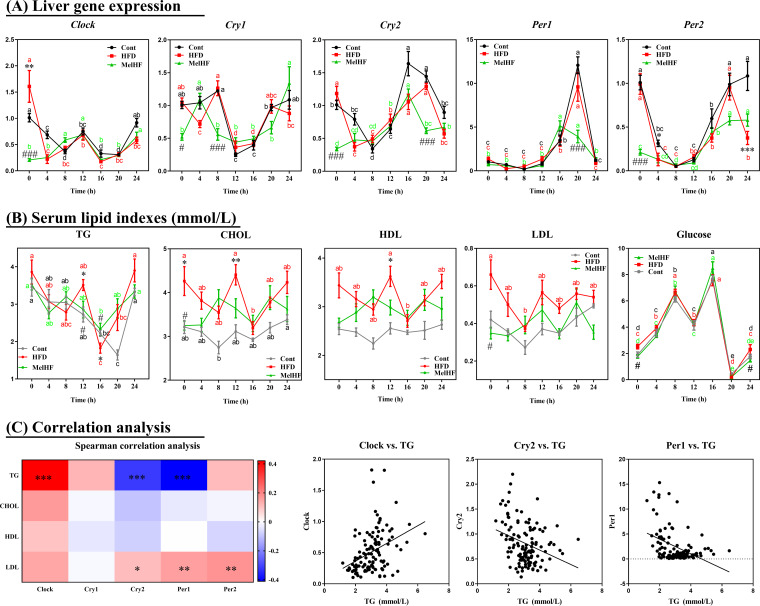
Effects of administration of exogenous melatonin on the diurnal rhythmicity of liver clock gene mRNA (*Clock*, *Cry1*, *Cry2*, *Per1*, and *Per2*) and serum lipid levels (TG, CHOL, HDL, LDL, and glucose) in HFD-fed mice. Liver gene expression (A), serum lipid levels (B), and correlation analysis between circadian clock genes and serum lipid indexes (C). Gene expression was determined by real-time PCR analysis, and relative gene expression levels were normalized to those of β-actin. Values are presented as the means ± SEMs. Differences between groups were assessed by Bonferroni’s test and denoted as follows: */#, *P* < 0.05; ***/###, *P* < 0.001. * indicates the difference between the control and HFD groups, whereas # indicates the difference between the HFD and MelHF groups. Spearman’s correlation analysis was conducted, and the correlation coefficient was used for the heat map: ***, *P* < 0.001. Multivariate analysis of variance for the time series was conducted by Duncan’s test, and values with different lowercase letters (a, b, c, and d) are significantly different (*P* < 0.05).

The circadian clock and metabolism are generally impaired in HFD-fed mice (27, 32). Thus, we further analyzed the diurnal variation in circadian clock genes (*Clock*, *Cry1*, *Cry2*, *Per1*, and *Per2*) in response to HFD and administration of exogenous melatonin (Fig. 2A; Table 1). Interestingly, *Clock* mRNA showed significant rhythmicity in the livers of control (*P* < 0.01) and MelHF (*P* < 0.05) mice, but not in the HFD group (*P* > 0.05), whereas the expression of *Cry1*, *Cry2*, *Per1*, and *Per2* in the liver showed a significant daily rhythm in all groups (*P* < 0.05).

**TABLE 1:** Mesor, amplitude, and acrophase of mRNA levels of clock genes in the livers of control, HFD, and MelHF mice^*a*^

Gene	Group	Acrophase (h)	Mesor	Amplitude	*P* value
*Clock*	Cont	0.59	0.61	0.21	<0.01
	HFD				ns
	MelHF	6.57	0.41	0.16	<0.05
*Cry1*	Cont	1.90	0.85	0.37	<0.01
	HFD	1.12	0.82	0.27	<0.01
	MelHF	0.54	0.71	0.27	<0.01
*Cry2*	Cont	20.42	0.97	0.56	<0.01
	HFD	20.63	0.83	0.40	<0.01
	MelHF	17.84	0.64	0.30	<0.01
*Per1*	Cont	19.60	2.88	4.20	<0.01
	HFD	19.30	2.44	3.30	<0.01
	MelHF	18.38	1.90	2.37	<0.01
*Per2*	Cont	22.27	0.60	0.5	<0.01
	HFD	21.91	0.44	0.38	<0.01
	MelHF	20.89	0.30	0.25	<0.01

a The rhythmicity was assessed by cosinor analysis, and *P* < 0.05 indicated a significant rhythm; ns means the difference was nonsignificant (*P* > 0.05). The model can be written according to the equation f(x) = A + B cos [2 π(x + C)/24], with f(x) indicating relative expression levels of target genes, x indicating the time of sampling (h), A indicating the mean value of the cosine curve (midline estimating statistic of rhythm [mesor]), B indicating the amplitude of the curve (half of the sinusoid), and C indicating the acrophase (h).

### Diurnal rhythms of serum lipids in response to HFD and exogenous melatonin.

Next, we determined the diurnal patterns of serum lipids and glucose in the three experimental groups (Fig. 2B; Table 2). Serum triglycerides (TG) exhibited significant rhythmicity in control and MelHF mice (*P* < 0.01) but not in HFD mice (*P* > 0.05). A significant diurnal rhythm of low-density lipoprotein (LDL) was observed in only control mice (*P* < 0.01). Serum glucose exhibited rhythmicity in all three groups (*P* < 0.01). No daily rhythms were observed in the levels of serum cholesterol (CHOL) and high-density lipoprotein (HDL) (*P* > 0.05). Despite the rhythmicity, overall lipid indexes were very high in HFD-fed mice, while the trends in the MelHF group were similar to those of control subjects, and the values were much lower than those for the HFD-fed mice at specific time points, as previously shown (17).

**TABLE 2:** Mesor, amplitude, and acrophase of serum lipid indexes^*a*^

Item	Group	Acrophase (h)	Mesor	Amplitude	*P* value
TG	Cont	0.79	2.96	0.75	<0.01
	HFD				ns
	MelHF	0.62	3.13	0.45	<0.01
CHOL	Cont				ns
	HFD				ns
	MelHF				ns
HDL	Cont				ns
	HFD				ns
	MelHF				ns
LDL	Cont	23.2	0.39	0.06	<0.01
	HFD				ns
	MelHF				ns
Glucose	Cont	11.56	3.56	2.87	<0.01
	HFD	10.47	3.72	2.93	<0.01
	MelHF	11.5	3.98	2.89	<0.01

a The rhythmicity was assessed by cosinor analysis, and *P* < 0.05 indicated a significant rhythm; ns means the difference was nonsignificant (*P* > 0.05). The model can be written according to the equation f(x) = A + B cos [2 π(x + C)/24], with f(x) indicating relative expression levels of target genes, x indicating the time of sampling (h), A indicating the mean value of the cosine curve (mesor; midline estimating statistic of rhythm [mesor]), B indicating the amplitude of the curve (half of the sinusoid), and C indicating the acrophase (h).

To determine whether serum lipid rhythmicity was associated with the liver expression of clock genes, we performed Pearson correlation analysis among serum lipid indexes and circadian clock genes (*Clock*, *Cry1*, *Cry2*, *Per1*, and *Per2*) (Fig. 2C). Surprisingly, serum TG concentration was positively correlated with *Clock* expression but exhibited a negative correlation with the mRNA levels of *Cry2* and *Per1* (*P* < 0.001). Together, the rhythmicity of lipid indexes, especially TG concentration, was widely observed in the blood and was markedly associated with clock gene expression. The daily rhythm of TG was impaired in the HFD-fed mice, which was markedly improved by administration of exogenous melatonin.

### Effect of melatonin on the diurnal rhythms of the gut microbiota in HFD-fed mice.

The gut microbiota has been identified as a key element involved in host circadian rhythms and itself also undergoes circadian oscillation, which is disturbed in HFD-fed mice or obesity models (27, 28, 33). Our previous study demonstrated that melatonin treatment improved lipid metabolism by reprogramming the gut microbiota in HFD-fed mice (17); thus, we hypothesized that administration of exogenous melatonin would improve the daily rhythm of the gut microbiota.

Mice were sacrificed every 4 h within a 24-h period, and metagenomic DNA was extracted from the cecal contents. The gut microbiota was tested by 16S rRNA gene sequencing, and the compositions were similar to those observed in our previous study (17), that is, the most abundant phylum, *Bacteroidetes*, was decreased in HFD-fed mice, and the abundance of *Firmicutes* increased; melatonin reversed these alterations (see Fig. S1 in the supplemental material). *Firmicutes* exhibited significant rhythmicity in control and MelHF mice (*P* < 0.05) but not in HFD mice (*P* > 0.05), while *Bacteroidetes* exhibited rhythmicity in only the control and HFD groups (*P* < 0.05) (Table 3). The relative abundance of *Firmicutes* peaked at 4:00 in the HFD group but at 8:00 in the control and MelHF groups (Fig. S1). However, *Proteobacteria* and *Actinobacteria* failed to show a diurnal variation at the phylum level (Table 3).

**TABLE 3:** Mesor, amplitude, and acrophase of gut microbiota compositions^*a*^

Group(s)	Acrophase (h)	Mesor	Amplitude	*P* value
Microbiota at the phylum level				
*Firmicutes*				
Cont	5.37	0.37	0.13	<0.05
HFD				ns
MelHF	6.05	0.34	0.14	<0.05
*Bacteroidetes*				
Cont	22.40	0.59	0.12	<0.05
HFD	22.01	0.55	0.11	<0.05
MelHF				ns
*Proteobacteria* and *Actinobacteria*				
Cont, HFD, and MelHF				ns
Microbiota at the genus level				
*Bacteroides* and *Desulfovibrio*				
Cont, HFD, and MelHF				ns
*Parasutterella*				
Contr	19.28	0.01	0.01	<0.05
HFD and MelHF				ns
*Ruminococcacea*				ns
Cont and HFD				ns
MelHF	1.13	0.009	0.003	<0.05
*Oscillibacter*				
Cont	2.92	0.002	0.002	<0.01
HFD	2.48	0.0004	0.003	<0.01
MelHF				ns
*Rikenella*				
Cont	0.35	0.006	0.003	<0.05
HFD	2.19	0.005	0.003	<0.05
MelHF				ns
*Lachnoclostridium*				
Cont	4.73	0.005	0.003	<0.05
HFD	3.17	0.005	0.003	<0.05
MelHF				ns
*Anaerotruncus*				
Cont	5.70	0.002	0.00004	<0.01
HFD				ns
MelHF	3.35	0.003	0.002	<0.01
*Lactobacillus*				
Cont	12.77	0.22	0.11	<0.05
HFD	13.98	0.16	0.12	<0.01
MelHF	12.85	0.17	0.17	<0.01
*Alloprevotella*				
Cont	23.59	0.05	0.03	<0.01
HFD and MelHF				ns
*Helicobacter*				
Cont and HFD				ns
MelHF	3.33	0.02	0.03	<0.05
*Lachnospiraceae*				
Cont				ns
HFD	2.43	0.04	0.02	<0.05
MelHF	2.74	0.03	0.03	<0.05
*Intestinimonas*				
Cont and MelHF				ns
HFD	3.18	0.01	0.01	<0.01
*Parabacteroides*				ns
Cont	0.18	0.02	0.008	<0.01
HFD and MelHF				ns
*Ruminiclostridium*				
Cont	2.83	0.007	0.007	<0.05
HFD	1.59	0.01	0.008	<0.05
MelHF	1.17	0.007	0.005	<0.01
*Roseburia*				
Cont and HFD				ns
MelHF	3.68	0.002	0.002	<0.01
*Clostridiales*				
Cont, HFD, and MelHF				ns
*Alistipes*				
Cont	23.14	0.004	0.002	<0.01
HFD and MelHF				ns

a The rhythmicity was assessed by cosinor analysis, and *P* < 0.05 indicated a significant rhythm; ns means the difference was nonsignificant (*P* > 0.05). The model can be written according to the equation f(x) = A + B cos [2 π(x + C)/24], with f(x) indicating relative expression levels of target genes, x indicating the time of sampling (h), A indicating the mean value of the cosine curve (midline estimating statistic of rhythm [mesor]), B indicating the amplitude of the curve (half of the sinusoid), and C indicating the acrophase (h).

10.1128/mSystems.00002-20.1 FIG S1 Administration of exogenous melatonin improved the composition and diurnal rhythmicity of the gut microbiota in HFD-fed mice. Microbiota compositions at the phylum level (A), *Firmicutes* (B), *Bacteroidetes* (C), and oscillating phyla (D). Values are presented as the means ± SEMs. Differences between groups were assessed by Bonferroni’s test and denoted as follows: *^/#^, *P* < 0.05. * indicates the difference between the control and HFD groups; # indicates the difference between the HFD and MelHF groups. Multivariate analysis of variance for the time series was conducted by Duncan’s test, and values with different lowercase letters are significantly different (*P* < 0.05). Download FIG S1, TIF file, 1.2 MB. Copyright © 2020 Yin et al. 2020 Yin et al. This content is distributed under the terms of the Creative Commons Attribution 4.0 International license.

**FIG 3:**
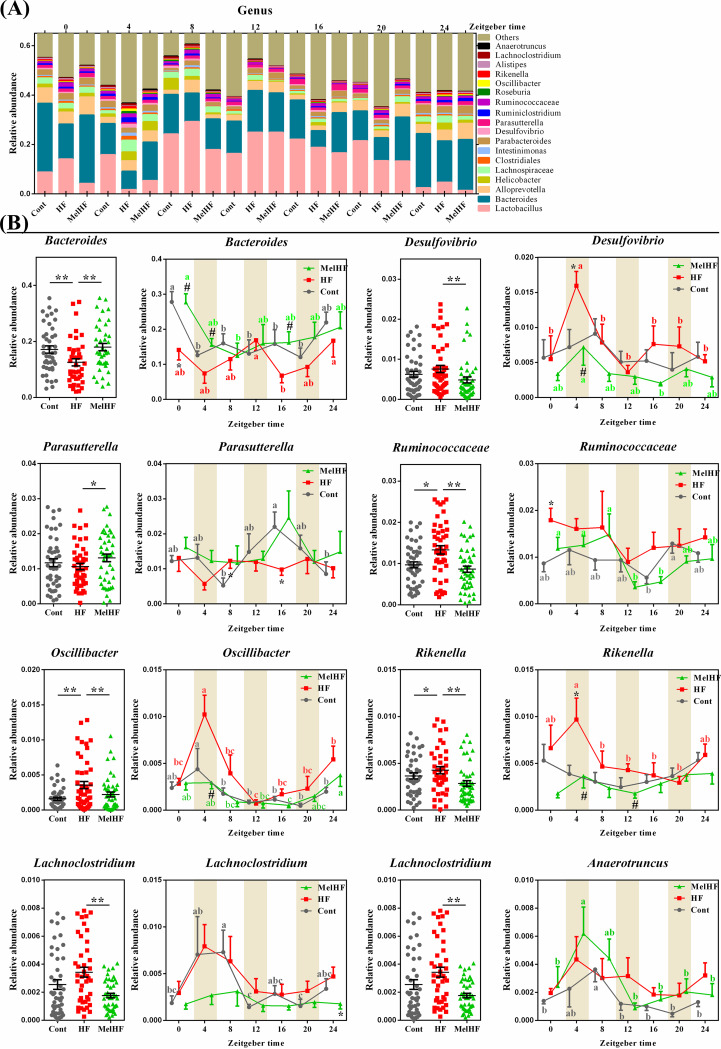
Administration of exogenous melatonin improved the composition and diurnal rhythmicity of the gut microbiota in HFD-fed mice. Microbiota compositions at the genus level (A) and microbiota compositions and oscillating genera (B). Values are presented as the means ± SEMs. Differences between groups were assessed by Bonferroni’s test and denoted as follows: */#, *P* < 0.05. * indicates the difference between the control and HFD groups; # indicates the difference between the HFD and MelHF groups. Multivariate analysis of variance for the time series was conducted by Duncan’s test, and values with different lowercase letters (a, b, and c) are significantly different (*P* < 0.05).

At the genus level, 8 genera were significantly altered, and most of them exhibited a marked daily rhythmicity, except for *Bacteroides*, *Desulfovibrio*, and *Clostridiales* (*P* > 0.05) (Fig. 3; Table 3; see also Fig. S2). *Parasutterella* (*P* < 0.05), *Alloprevotella* (*P* < 0.01), *Parabacteroides* (*P* < 0.01), and *Alistipes* (*P* < 0.01) were only rhythmic in the control group. *Intestinimonas* exhibited a significant rhythm in only HFD-fed mice (*P* < 0.01). *Ruminococcaceae* (*P* < 0.05), *Helicobacter* (*P* < 0.05), and *Roseburia* (*P* < 0.01) showed a daily rhythm in only the melatonin-treated mice. *Oscillibacter*, *Rikenella*, and *Lachnoclostridium* exhibited marked cycles in the control and HFD groups (*P* < 0.05) but not in the MelHF group (*P* > 0.05). *Anaerotruncus* showed a diurnal pattern in the control and MelHF groups (*P* < 0.01) but not in HFD-fed mice (*P* > 0.05). We also noticed that *Lachnospiraceae* was rhythmic in the HFD and MelHF groups (*P* < 0.05) but not in the control group (*P* > 0.05). In addition, *Lactobacillus* and *Ruminiclostridium* exhibited rhythmicity regardless of HFD and melatonin challenges (*P* < 0.05).

10.1128/mSystems.00002-20.2 FIG S2 Oscillating genera. Values are presented as the means ± SEMs. Differences between groups were assessed by Bonferroni’s test and denoted as follows: *^/#^, *P* < 0.05. * indicates the difference between the control and HFD groups; # indicates the difference between the HFD and MelHF groups. Multivariate analysis of variance for the time series was conducted by Duncan’s test, and values with different lowercase letters are significantly different (*P* < 0.05). Download FIG S2, TIF file, 3.0 MB. Copyright © 2020 Yin et al. 2020 Yin et al. This content is distributed under the terms of the Creative Commons Attribution 4.0 International license.

Collectively, our data showed that most of the microbiota exhibited daily variation and that the diurnal network of some gut microbiota was affected by HFD and reversed, at least in part, by administration of exogenous melatonin (Fig. S2).

### Genome prediction of microbial communities.

The gut microbiota has a widespread and modifiable effect on host gene regulation (34); thus, metabolism, genetic information, environmental information, cellular processes, human diseases, and organismal system pathways were further annotated according to the microbiota compositions by Tax4Fun analysis (see Fig. S4A). Our data show that short-term HFD feeding markedly affected cell growth and death, endocrine and metabolic diseases, the endocrine system, the nervous system, the immune system, and environmental adaptation (*P* < 0.05), while administration of exogenous melatonin influenced lipid metabolism, terpenoids, and polyketides (*P* < 0.05). We then further analyzed lipid metabolism (Fig. S4B) and identified eight pathways that mainly contributed lipid metabolism-annotated genes, namely, lipid biosynthesis, fatty acid biosynthesis, glycerophospholipid metabolism, glycerolipid metabolism, sphingolipid metabolism, fatty acid degradation, biosynthesis of unsaturated fatty acids, and synthesis and degradation of ketone bodies (Fig. S4C).

10.1128/mSystems.00002-20.3 FIG S3 Microbial network. Download FIG S3, TIF file, 1.4 MB. Copyright © 2020 Yin et al. 2020 Yin et al. This content is distributed under the terms of the Creative Commons Attribution 4.0 International license.

10.1128/mSystems.00002-20.4 FIG S4 Predictive functional profiling of microbial communities by Tax4Fun analysis. KEGG pathway annotations (A), lipid metabolism (B), and detailed analysis of lipid metabolic pathways (C). Differences were assessed by Bonferroni’s test and denoted as follows: *^/#^, *P* < 0.05; **^/##^, *P* < 0.01. * indicates the difference between the control and HFD groups; # indicates the difference between the HFD and MelHF groups. Download FIG S4, TIF file, 2.2 MB. Copyright © 2020 Yin et al. 2020 Yin et al. This content is distributed under the terms of the Creative Commons Attribution 4.0 International license.

### Gut microbes correlated with clock genes and serum lipid levels.

**FIG 4:**
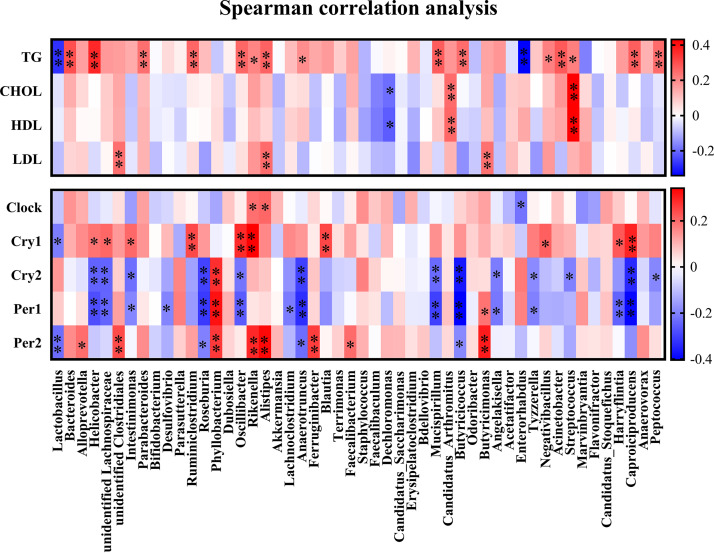
Correlation analysis of gut microbiota between clock gene expression and serum lipid levels. Spearman’s correlation analysis was conducted, and the correlation coefficient was used for the heat map: *, *P* < 0.05; **, *P* < 0.01.

We then investigated whether the gut microbiota (top 50) also showed an association with clock gene expression and serum lipid levels by Spearman’s test (Fig. 4A). The relative abundances of *Rikenella*, *Alistipes*, and *Enterorhabdus* were positively correlated with *Clock* mRNA (*P* < 0.05) (Fig. 4). Ten genera (i.e., *Helicobacter*, unidentified *Lachnospiraceae*, *Intestinimonas*, *Ruminiclostridium*, *Oscillibacter*, *Rikenella*, *Blautia*, *Negativibacillus*, *Harryflintia*, and *Caproiciproducens*) showed a positive association with *Cry1* mRNA (*P* < 0.05), while the correlation was negative between *Cry2* mRNA and most genera, such as *Helicobacter*, unidentified *Lachnospiraceae*, *Intestinimonas*, *Roseburia*, *Oscillibacter*, *Anaerotruncus*, *Mucispirillum*, *Butyricicoccus*, *Angelakisella*, *Tyzzerella*, *Streptococcus*, *Caproiciproducens*, and *Peptococcus*. The expressions of *Per1* and *Per2* shared the markedly correlation to the relative abundances of *Roseburia*, *Phyllobacterium*, *Anaerotruncus*, *Butyricicoccus*, and *Butyricimonas*. Together, 29 genera were found to be correlated with clock gene expression; these correlations were mostly positive with *Clock*, *Cry1*, and *Per2* mRNA and negative with *Cry2* and *Per1* mRNA.

A correlation analysis between serum lipid indexes and the gut microbiota was further conducted, and 17 genera (34% of top 50) were observed to be markedly correlated with TG concentrations (Fig. 4), including *Lactobacillus*, *Bacteroides*, *Helicobacter*, *Parabacteroides*, *Ruminiclostridium*, *Oscillibacter*, *Rikenella*, *Alistipes*, *Anaerotruncus*, *Mucispirillum*, *Butyricicoccus*, *Enterorhabdus*, *Negativibacillus*, Acinetobacter, *Streptococcus*, *Caproiciproducens*, and *Peptococcus*. The relative abundances of *Dechloromonas*, “*Candidatus* Arthromitus,” and *Streptococcus* showed significant correlations with both TG and HDL levels, while negative correlations were noticed between LDL and unidentified *Clostridiale*s, *Alistipes*, and *Butyricimonas*.

### Effects of exogenous melatonin during daytime or nighttime on lipid accumulation in HFD-fed mice.

**FIG 5:**
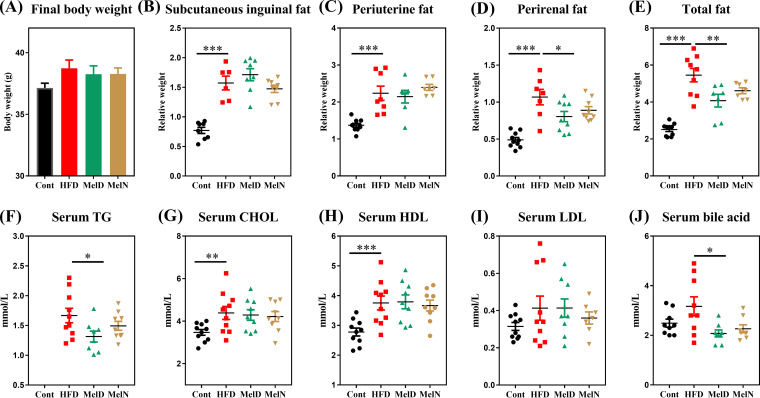
Effects of melatonin treatment during daytime and nighttime on lipid accumulation in HFD-fed mice. Final body weights (A), relative weights of subcutaneous inguinal fat (B), relative weights of periuterine fat (C), relative weights of perirenal fat (D), relative weights of total fat (E), serum TG concentrations (F), serum CHOL concentrations (G), serum HDL concentrations (H), serum LDL concentrations (I), and serum bile acid concentrations (J). Values are presented as the means ± SEMs. Differences between groups were assessed by Bonferroni’s test and denoted as follows: *, *P* < 0.05; **, *P* < 0.01; ***, *P* < 0.001.

We further determined the effect of melatonin treatment during daytime or nighttime on lipid metabolism and the gut microbiota. HFD-fed mice showed high relative weights of subcutaneous inguinal fat, periuterine fat, perirenal fat, and total fat (*P* < 0.001) (Fig. 5A to E). Administration of exogenous melatonin during daytime markedly reduced perirenal fat (*P* < 0.05) and total fat (*P* < 0.01) weights (Fig. 5D and E), but the trend was nonsignificant for the nighttime treatment compared with the control group (*P* > 0.05) (Fig. 5B to E). We also tested serum lipid indexes (Fig. 5F to J), and the results showed that serum TG and bile acid concentrations were markedly reduced in the daytime melatonin (MelD) group (*P* < 0.05) but not in the nighttime melatonin (MelN) group (*P* > 0.05). Taken together, we failed to notice any significant difference in host lipid metabolism between daytime and nighttime melatonin exposure.

**FIG 6:**
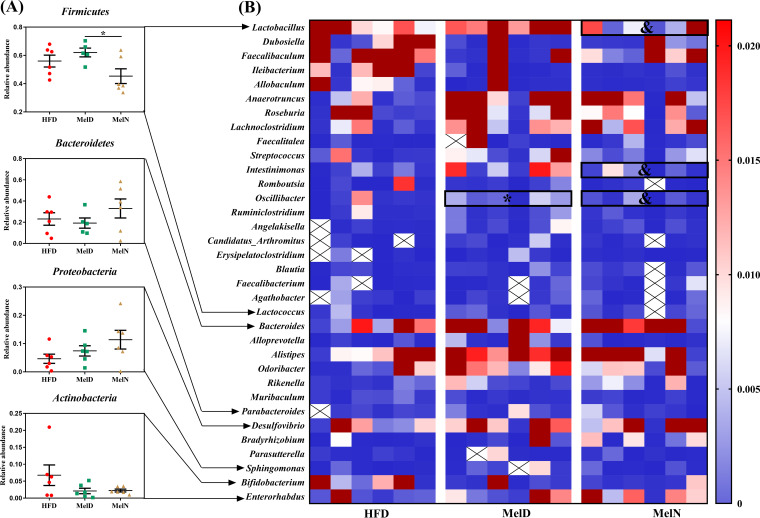
Melatonin treatment during daytime and nighttime had different effects on gut microbiota compositions in HFD-fed mice. The microbiota at the phylum (A) and genus (B) levels. Values are presented as the means ± SEMs. Differences between groups were assessed by Bonferroni’s test and denoted as follows: */&, *P* < 0.05; * indicates the difference was significant compared with the HFD group; & indicates the difference was significant between the MelD and MelN groups.

We then investigated the gut microbiota compositions of the HFD, MelD, and MelN groups using 16S rRNA gene sequencing. At the phylum level, melatonin treatment during daytime or nighttime failed to alter the gut microbiota composition (Fig. 6A). Interestingly, administration of exogenous melatonin during nighttime significantly reduced the relative abundance of *Firmicutes* compared with that for the daytime treatment (*P* < 0.05). At the genus level, *Lactobacillus*, *Intestinimonas*, and *Oscillibacter* were significantly affected by melatonin treatment during the day or the night (*P* < 0.05) (Fig. 6B).

### Microbiota transplantation at different times of the day affected lipid metabolism in HFD-fed mice.

**FIG 7:**
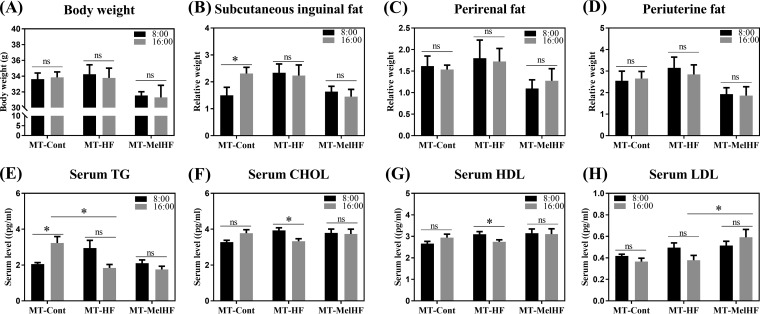
Microbiota transplantation at different times of the day affected lipid metabolism in HFD-fed mice. Final body weights (A), relative weights of subcutaneous inguinal fat (B), relative weights of perirenal fat (C), relative weights of periuterine fat (D), serum TG concentrations (E), serum CHOL concentrations (F), serum HDL concentrations (G), and serum LDL concentrations (H). The black bars indicate the fecal microbiota transplanted at 8:00, while the gray bars indicate transplantation at 16:00. Values are presented as the means ± SEMs. Differences between 8:00 and 16:00 in one group were assessed by Student's *t* test, and multiple comparisons between groups (MT-Cont, MT-HF, and MT-MelHF) were analyzed by Bonferroni’s test and denoted as follows: *, *P* < 0.05; ns, *P* > 0.05.

As gut microbiota correlated with serum lipids and both gut microbiota and serum lipid indexes exhibited a daily rhythmicity, which is highly driven by HFD feeding and melatonin drinking, we next performed fecal microbiota transplantation at two different time points (8:00 and 16:00) from the control, HFD, and MelHF groups into antibiotic-treated mice to investigate the response to HFD feeding. Body weights were recorded, and no significant difference was observed between the two time points (Fig. 7A to D). Interestingly, the relative weight of subcutaneous inguinal fat in the group receiving transplants from controls (MT-Cont group) was affected by the time at which the microbiota was transplanted (*P* < 0.05).

Similar to the results of our previous study (17), microbiota transplantation at 8:00 from the HFD group tended to enhance serum TG, CHOL, and HDL concentrations, which were slightly reversed in the group receiving transplants from MelHF mice (MT-MelHF group) (Fig. 7E to G). Conversely, serum TG concentration was reduced in the MT-HF group (*P* < 0.05) when microbiota transplantation was performed at 16:00 (Fig. 7E), and LDL was increased in the MT-MelHF group (*P* < 0.05) (Fig. 7H). Notably, microbiota transplantation from control subjects at 8:00 tended to enhance serum CHOL and HDL (*P* > 0.05) (Fig. 7F and G) and significantly increased TG concentrations (*P* < 0.05) (Fig. 7E) compared with those after microbiota transplantation at 16:00 (Fig. 7B). However, serum CHOL and HDL levels were lower at 16:00 than at 8:00 for HFD-derived microbiota transplantation (*P* < 0.05) (Fig. 7E to G). No difference was observed between the two time points in the MT-MelHF group.

## DISCUSSION

We previously showed that administration of exogenous melatonin improves HFD-induced lipid metabolic disorder by reversing the gut microbiota composition, especially in terms of the relative abundances of *Firmicutes* and *Bacteroidetes* (17). Here, we further confirmed that melatonin may reverse the gut microbiota composition in HFD-fed mice and that the gut microbiota is closely associated with circadian clock genes and serum lipid indexes.

Diurnal rhythms and metabolism are tightly linked, and obesity leads to profound reorganization of the circadian system, leading to remodeling of the coordinated oscillations between associated transcripts and metabolites (35). For example, 38 metabolites and 654 transcripts were identified to be oscillating in only HFD-fed animals, and a majority of oscillations were clock dependent (35, 36). In this study, circadian clock genes (*Clock*, *Cry1*, *Cry2*, *Per1*, and *Per2*) and serum TG, LDL, and glucose concentrations exhibited daily rhythmicity, which is similar to the results of previous studies showing that most circadian genes are rhythmic in the liver (37). Interestingly, *Clock* and TG only cycled in the control and MelHF groups but not in the HFD-fed mice, indicating that daily rhythmicity was impaired by short-term HFD feeding, and administration of exogenous melatonin partially rescued the daily rhythmicity in HFD-fed mice. Strikingly, the serum TG concentration was positively correlated with *Clock* mRNA and negatively correlated with *Cry2* and *Per1* mRNA.

Compelling experimental evidence has shown a marked difference in the gut microbiota between obese and lean subjects (38–40). Here, we further investigated the correlation between the microbiota (at the genus level) and the circadian clock genes and serum lipid levels. Fourteen genera showed a significant correlation with clock gene expression. Positive correlations were observed with *Clock* and *Cry1* mRNA levels, and negative correlations were observed with *Cry2* and *Per1*. Notably, *Alloprevotella* and *Rikenella* were found to be associated with *Clock*, *Cry1*, and *Per2*, whereas *Helicobacter* and *Anaerotruncus* were correlated with *Cry2*, *Per1*, and *Per2*. Previous studies have reported that germfree mice show reduced amplitudes of clock gene expression in both central and peripheral tissues even in the presence of light-dark signals (27). Taken together, our data may further indicate that the diurnal variations in clock genes may be governed, at least in part, by the gut microbiota. In addition, *Lactobacillus*, *Bacteroides*, *Helicobacter*, *Parabacteroides*, *Ruminiclostridium*, *Rikenella*, and *Alistipes* were correlated with serum TG, and *Rikenella*, *Alistipes*, and *Clostridiales* were closely associated with LDL concentration. Among these genera, *Lactobacillus* has been extensively studied and has been shown to be involved in lipid accumulation (41–43), which is markedly enhanced in HFD-fed mice and reversed by administration of melatonin (17). Our previous study indicated that *Bacteroides-* and *Alistipes*-derived acetic acids target host lipid metabolism (17), which is further corroborated by the present data showing that both *Bacteroides* and *Alistipes* were markedly associated with serum TG or LDL.

Microbiota analysis within a 24-h period further confirmed that administration of melatonin reverses the gut microbiota composition, especially in terms of the relative abundances of *Firmicutes* and *Bacteroidetes* (16, 17). In addition, we have also shown that most gut microbes exhibit daily cyclical variation under a variety of dietary and melatonin treatments (26–28, 33). However, the diurnal variations in the gut microbiota are highly variable. For example, *Firmicutes* cycled in control and MelHF mice (*P* < 0.05) but not in HFD mice (*P* > 0.05). Additionally, the *Firmicutes* abundance in HFD-fed mice peaked at 4:00 and was markedly different from the abundances in the control and MelHF groups, in which the *Firmicutes* abundance peaked at 8:00. Conversely, HFD mice exhibited the lowest abundance of *Bacteroidetes* at 4:00 compared with that at 8:00 in the control and MelHF groups. At the genus level, we also show that most genera oscillate within a 24-h period and that the cosine curves of the microbiota are similar between the control and MelHF groups, suggesting that the daily rhythm of the gut microbiota is driven by HFD and reversed by melatonin administration. Microbiota rhythms have been indicated to represent a potential mechanism by which the gut microbiota affects host metabolism (26). Using a germfree animal model, Thaiss et al. found that microbiota deficiency leads to a temporal reorganization of metabolic pathways, as evidenced by the reduction in chromatin and transcript oscillations and the substantial increase in *de novo* oscillations (44). Taken together, our new data support the hypothesis that the diurnal rhythmicity of the gut microbiota in HFD-fed mice is improved by administration of exogenous melatonin, while the role by directly targeting gut microbiota or indirectly modulation of body weight and lipid metabolism should be further studied.

Another important finding from the present study is that melatonin treatment during daytime, but not nighttime, markedly improved HFD-induced lipid dysmetabolism. The underlying reason may be associated with the secretory mechanism, that is, melatonin is mainly secreted at night, and melatonin treatment during daytime leads to the maintenance of a high level of melatonin, providing sustained exposure of host metabolism to melatonin (45). Our previous study showed that *Lactobacillus* is enriched in HFD-fed mice, which is reversed by administration of melatonin (17). Similarly, the relative abundance of *Oscillibacter* is greatly increased in HFD-fed mice (46, 47), indicating a potential role of *Lactobacillus* and *Oscillibacter* in the melatonin-mediated lipid metabolic response. Microbiota transplantation from different groups at different times led to different susceptibilities to HFD-induced lipid dysmetabolism, which further demonstrates the diurnal rhythmicity of the gut microbiota. Notably, serum lipid indexes show a marked difference between the two time points of microbiota transplantation from control and HFD mice but not from melatonin-treated animals, indicating that the diurnal alteration of gut microbiota is affected by melatonin treatment.

### Conclusion.

In conclusion, our results show that most gut microbes exhibit a daily rhythm and are closely associated with clock gene expression and serum lipid levels. Melatonin improves the diurnal patterns of the gut microbiota in HFD-fed mice, which is further confirmed by microbiota transplantation. Microbiota transplantation early in the morning or in the late afternoon also lead to diverse responses to HFD. Taken together, we conclude that most gut microbiota cycles occur within a 24-h period, and the rhythm is disturbed by HFD feeding, while administration of exogenous melatonin improves diurnal patterns of some specific microbiota in HFD-fed mice. However, the detailed mechanisms behind melatonin mediated-gut microbiota and metabolic rhythmicity (directly targeted or indirect modulation of body weight) have not be fully resolved; thus, melatonin treatment in a healthy model and a 48-h rhythmic analysis are suggested to confirm the merit of melatonin in obesity.

## MATERIALS AND METHODS

### Animals and diet.

ICR mice, a melatonin-deficient strain, were used in this study to eliminate the effect of endogenous melatonin production (SLAC Laboratory Animal Central, Changsha, China). As sex affects the melatonin profile (48), only female mice were used in this study to rule out this effect. All animals had free access to food and drinking water (temperature, 25 ± 2°C; relative humidity, 45% to 60%; lighting cycle, 12 h/day) during the experiment. The diets used in this study were as described in our previous study (17).

### Melatonin treatment.

A total of 126 female mice (22.77 ± 0.10 g, approximately 4 weeks old) were randomly grouped into the control (Cont), HFD, and HFD plus melatonin (MelHF) groups (*n* = 42). Mice in the MelHF group received the HFD and melatonin-containing water (0.4 mg/ml melatonin [Meilun, Dalian, China], directly diluted in drinking water) (17). The melatonin solution was prepared daily and kept in a normal bottle with an aluminum foil cover to prevent light-induced degradation of melatonin. After 2 weeks of melatonin administration, 6 mice in each group were randomly killed at 0:00 (Zeitgeber time 16 [ZT16]), 4:00 (ZT20), 8:00 (ZT0, lights on), 12:00 (ZT4), 16:00 (ZT8), 20:00 (ZT12, lights off), and 24:00 (ZT16) (*n* = 6). Blood samples were collected by orbital bleeding. Liver, adipose tissue, and colonic digesta samples were weighed and collected.

### Melatonin treatment during daytime and nighttime.

Mice (26.89 ± 0.15 g) were randomly grouped into a control and three HFD groups (*n* = 12). One group of HFD mice received melatonin during daytime (8:00 to 16:00) and control water at night (16:00 to 8:00) (MelD), and another received melatonin during nighttime (16:00 to 8:00) and control water during daytime (8:00 to 16:00) (MelN). All mice were sacrificed at 8:00 a.m. after 2 weeks of feeding, and samples were collected for further analyses.

### Fecal microbiota transplantation.

Mice were treated with antibiotics (1 g/liter streptomycin, 0.5 g/liter ampicillin, 1 g/liter gentamicin, and 0.5 g/liter vancomycin) to clear the gut microbiota (17). After 1 week of antibiotic treatment, the antibiotic-containing water was replaced with regular water, and the microbiota-depleted mice received transplants of the donor microbiota. Fecal supernatants from the control (MT-Cont), HFD (MT-HF), and MelHF (MT-MelHF) (treated for 14 days) mice were transplanted into the microbiota-depleted mice at 8:00 and 16:00 (for 5 days). Following transplantation, all mice further received HFD and regular water for an additional 14 days.

### Serum lipid indexes.

Serum samples were separated after centrifugation at 3,000 rpm for 10 min at 4°C. A Cobas c-311 Coulter chemistry analyzer was used to test serum biochemical parameters (17, 49), including triglycerides (TG), cholesterol (CHOL), high-density lipoprotein (HDL), low-density lipoprotein (LDL), glucose, and bile acid, as these indexes are commonly dysregulated in HFD-fed or obese subjects (50–53).

### Reverse transcription-PCR.

Liver samples were frozen in liquid nitrogen and ground, and total RNA was isolated by using TRIzol reagent (Invitrogen, USA) and then treated with DNase I (Invitrogen, USA). Reverse transcription was conducted at 37°C for 15 min at 95°C for 5 s. The primers used in this study were designed according to the mouse sequence (see Table S1 in the supplemental material). β-Actin was chosen as the housekeeping gene to normalize target gene levels. PCR cycling and relative expression determination were performed according to previous studies (54–61).

10.1128/mSystems.00002-20.5 TABLE S1 Primers used in this study. Download Table S1, DOCX file, 0.1 MB. Copyright © 2020 Yin et al. 2020 Yin et al. This content is distributed under the terms of the Creative Commons Attribution 4.0 International license.

### Microbiota profiling.

Total genome DNA from colonic samples was extracted for amplification using a specific primer with a barcode (16S V3+V4). Sequencing libraries were generated and analyzed according to our previous study (54, 62, 63). Operational taxonomic units (OTUs) were further used for genomic prediction of microbial communities by Tax4Fun analysis (64).

### Statistical analysis.

All statistical analyses were performed using one-way analysis of variance, and multiple comparisons were further conducted using Bonferroni analysis (SPSS 21 software). Spearman’s correlation analysis was conducted. The rhythmicity of clock genes, serum lipid indexes, and the gut microbiota was assessed by cosinor analysis using the nonlinear regression model within Sigmaplot V 10.0 (Systat Software, San Jose, CA, USA) (65). Multivariate analysis of variance for the time series was conducted by Duncan’s test, and values with different lowercase letters in the figure panels are significantly different. The data are expressed as the means ± standard errors of the means (SEMs). A *P* value of <0.05 was considered significant. All figures in this study were drawn by using GraphPad Prism 7.04.

### Data availability.

Raw sequences are available in the NCBI Sequence Read Archive with accession numbers SAMN11246274, PRJNA528844, SAMN11245315, and PRJNA528812.

## References

[1] Tordjman S, Chokron S, Delorme R, Charrier A, Bellissant E, Jaafari N, Fougerou C 2017 Melatonin: pharmacology, functions and therapeutic benefits. Curr Neuropharmacol 15:434–443. doi:10.2174/1570159X14666161228122115. 28503116 PMC5405617

[2] Claustrat B, Leston J 2015 Melatonin: physiological effects in humans. Neurochirurgie 61:77–84. doi:10.1016/j.neuchi.2015.03.002. 25908646

[3] Keijzer H, Smits MG, Duffy JF, Curfs L 2014 Why the dim light melatonin onset (DLMO) should be measured before treatment of patients with circadian rhythm sleep disorders. Sleep Med Rev 18:333–339. doi:10.1016/j.smrv.2013.12.001. 24388969

[4] Reiter RJ, Tamura H, Tan DX, Xu XY 2014 Melatonin and the circadian system: contributions to successful female reproduction. Fertil Steril 102:321–328. doi:10.1016/j.fertnstert.2014.06.014. 24996495

[5] Slats D, Claassen J, Verbeek MM, Overeem S 2013 Reciprocal interactions between sleep, circadian rhythms and Alzheimer’s disease: focus on the role of hypocretin and melatonin. Ageing Res Rev 12:188–200. doi:10.1016/j.arr.2012.04.003. 22575905

[6] Zisapel N 2018 New perspectives on the role of melatonin in human sleep, circadian rhythms and their regulation. Br J Pharmacol 175:3190–3199. doi:10.1111/bph.14116. 29318587 PMC6057895

[7] Jilg A, Bechstein P, Saade A, Dick M, Li TX, Tosini G, Rami A, Zemmar A, Stehle JH 2019 Melatonin modulates daytime-dependent synaptic plasticity and learning efficiency. J Pineal Res 66:e12553. doi:10.1111/jpi.12553. 30618149 PMC6405292

[8] Najafi M, Salehi E, Farhood B, Nashtaei MS, Goradel NH, Khanlarkhani N, Namjoo Z, Mortezaee K 2019 Adjuvant chemotherapy with melatonin for targeting human cancers: a review. J Cell Physiol 234:2356–2372. doi:10.1002/jcp.27259. 30192001

[9] do Amaral FG, Cipolla-Neto J 2018 A brief review about melatonin, a pineal hormone. Arch Endocrinol Metab 62:472–479. doi:10.20945/2359-3997000000066. 30304113 PMC10118741

[10] Meng JF, Shi TC, Song S, Zhang ZW, Fang YL 2017 Melatonin in grapes and grape-related foodstuffs: a review. Food Chem 231:185–191. doi:10.1016/j.foodchem.2017.03.137. 28449995

[11] Buonfiglio D, Tchio C, Furigo I, Donato J, Baba K, Cipolla‐Neto J, Tosini G 2019 Removing melatonin receptor type 1 signaling leads to selective leptin resistance in the arcuate nucleus. J Pineal Res 67:e12580. doi:10.1111/jpi.12580. 30968433 PMC6687516

[12] Owino S, Sanchez-Bretano A, Tchio C, Cecon E, Karamitri A, Dam J, Jockers R, Piccione G, Noh HL, Kim T, Kim JK, Baba K, Tosini G 2018 Nocturnal activation of melatonin receptor type 1 signaling modulates diurnal insulin sensitivity via regulation of PI3K activity. J Pineal Res 64:e12462. doi:10.1111/jpi.12462. PMC5843510 29247541

[13] Ozkok E, Yorulmaz H, Ates G, Aksu A, Balkis N, Sahin O, Tamer S 2016 Amelioration of energy metabolism by melatonin in skeletal muscle of rats with LPS induced endotoxemia. Physiol Res 65:833–842. 27875899 10.33549/physiolres.933282

[14] McMullan CJ, Schernhammer ES, Rimm EB, Hu FB, Forman JP 2013 Melatonin secretion and the incidence of type 2 diabetes. JAMA 309:1388–1396. doi:10.1001/jama.2013.2710. 23549584 PMC3804914

[15] Patel R, Rathwa N, Palit SP, Ramachandran AV, Begum R 2018 Association of melatonin and MTNR1B variants with type 2 diabetes in Gujarat population. Biomed Pharmacother 103:429–434. doi:10.1016/j.biopha.2018.04.058. 29674279

[16] Xu PF, Wang JL, Hong F, Wang S, Jin X, Xue TT, Jia L, Zhai YG 2017 Melatonin prevents obesity through modulation of gut microbiota in mice. J Pineal Res 62:e12399. doi:10.1111/jpi.12399. 28199741

[17] Yin J, Li Y, Han H, Chen S, Gao J, Liu G, Wu X, Deng J, Yu Q, Huang X, Fang R, Li T, Reiter RJ, Zhang D, Zhu C, Zhu G, Ren W, Yin Y 2018 Melatonin reprogramming of gut microbiota improves lipid dysmetabolism in high-fat diet-fed mice. J Pineal Res 65:e12524. doi:10.1111/jpi.12524. 30230594

[18] Moossavi S, Azad MB 2019 Quantifying and interpreting the association between early-life gut microbiota composition and childhood obesity. mBio 10:e02787-18. doi:10.1128/mBio.02787-18. 30755514 PMC6372801

[19] Gomes AC, Hoffmann C, Mota JF 2018 The human gut microbiota: metabolism and perspective in obesity. Gut Microbes 9:308–325. doi:10.1080/19490976.2018.1465157. 29667480 PMC6219651

[20] Yildirimer CC, Brown KH 2018 Intestinal microbiota lipid metabolism varies across rainbow trout (*Oncorhynchus mykiss*) phylogeographic divide. J Appl Microbiol 125:1614–1625. doi:10.1111/jam.14059. 30074661

[21] Lacroix S, Pechereau F, Leblanc N, Boubertakh B, Houde A, Martin C, Flamand N, Silvestri C, Raymond F, Di Marzo V, Veilleux A 2019 Rapid and concomitant gut microbiota and endocannabinoidome response to diet-induced obesity in mice. mSystems 4:e00407-19. doi:10.1128/mSystems.00407-19. 31848310 PMC6918026

[22] Shan K, Qu H, Zhou K, Wang L, Zhu C, Chen H, Gu Z, Cui J, Fu G, Li J, Chen H, Wang R, Qi Y, Chen W, Chen YQ 2019 Distinct gut microbiota induced by different fat-to-sugar-ratio high-energy diets share similar pro-obesity genetic and metabolite profiles in prediabetic mice. mSystems 4:e00219-19. doi:10.1128/mSystems.00219-19. 31594827 PMC6787563

[23] Song B, Zhong YZ, Zheng CB, Li FN, Duan YH, Deng JP 2019 Propionate alleviates high-fat diet-induced lipid dysmetabolism by modulating gut microbiota in mice. J Appl Microbiol 127:1546–1555. doi:10.1111/jam.14389. 31325215

[24] Cao GT, Dai B, Wang KL, Yan Y, Xu YL, Wang YX, Yang CM 2019 *Bacillus licheniformis*, a potential probiotic, inhibits obesity by modulating colonic microflora in C57BL/6J mice model. J Appl Microbiol 127:880–888. doi:10.1111/jam.14352. 31211897

[25] de Mendoza D, Pilon M 2019 Control of membrane lipid homeostasis by lipid-bilayer associated sensors: a mechanism conserved from bacteria to humans. Prog Lipid Res 76:100996. doi:10.1016/j.plipres.2019.100996. 31449824

[26] Zarrinpar A, Chaix A, Yooseph S, Panda S 2014 Diet and feeding pattern affect the diurnal dynamics of the gut microbiome. Cell Metab 20:1006–1017. doi:10.1016/j.cmet.2014.11.008. 25470548 PMC4255146

[27] Leone V, Gibbons SM, Martinez K, Hutchison AL, Huang EY, Cham CM, Pierre JF, Heneghan AF, Nadimpalli A, Hubert N, Zale E, Wang YW, Huang Y, Theriault B, Dinner AR, Musch MW, Kudsk KA, Prendergast BJ, Gilbert JA, Chang EB 2015 Effects of diurnal variation of gut microbes and high-fat feeding on host circadian clock function and metabolism. Cell Host Microbe 17:681–689. doi:10.1016/j.chom.2015.03.006. 25891358 PMC4433408

[28] Wang YH, Kuang Z, Yu XF, Ruhn KA, Kubo M, Hooper LV 2017 The intestinal microbiota regulates body composition through NFIL3 and the circadian clock. Science 357:912–916. doi:10.1126/science.aan0677. 28860383 PMC5702268

[29] Marcinkevicius EV, Shirasu-Hiza MM 2015 Message in a biota: gut microbes signal to the circadian clock. Cell Host Microbe 17:541–543. doi:10.1016/j.chom.2015.04.013. 25974294

[30] Giles C, Takechi R, Lam V, Dhaliwal SS, Mamo J 2018 Contemporary lipidomic analytics: opportunities and pitfalls. Prog Lipid Res 71:86–100. doi:10.1016/j.plipres.2018.06.003. 29959947

[31] Paulose JK, Wright JM, Patel AG, Cassone VM 2016 Human gut bacteria are sensitive to melatonin and express endogenous circadian rhythmicity. PLoS One 11:e0146643. doi:10.1371/journal.pone.0146643. 26751389 PMC4709092

[32] Gachon F, Yeung J, Naef F 2018 Cross-regulatory circuits linking inflammation, high-fat diet, and the circadian clock. Genes Dev 32:1359–1360. doi:10.1101/gad.320911.118. 30385518 PMC6217737

[33] Murakami M, Tognini P, Liu Y, Eckel-Mahan KL, Baldi P, Sassone-Corsi P 2016 Gut microbiota directs PPAR-driven reprogramming of the liver circadian clock by nutritional challenge. EMBO Rep 17:1292–1303. doi:10.15252/embr.201642463. 27418314 PMC5007574

[34] Richards AL, Muehlbauer AL, Alazizi A, Burns MB, Findley A, Messina F, Gould TJ, Cascardo C, Pique-Regi R, Blekhman R, Luca F 2019 Gut microbiota has a widespread and modifiable effect on host gene regulation. mSystems 4:e00323-18. doi:10.1128/mSystems.00323-18. PMC6722422 31481602

[35] Eckel-Mahan KL, Patel VR, de Mateo S, Orozco-Solis R, Ceglia NJ, Sahar S, Dilag-Penilla SA, Dyar KA, Baldi P, Sassone-Corsi P 2013 Reprogramming of the circadian clock by nutritional challenge. Cell 155:1464–1478. doi:10.1016/j.cell.2013.11.034. 24360271 PMC4573395

[36] Eckel-Mahan KL, Patel VR, Mohney RP, Vignola KS, Baldi P, Sassone-Corsi P 2012 Coordination of the transcriptome and metabolome by the circadian clock. Proc Natl Acad Sci U S A 109:5541–5546. doi:10.1073/pnas.1118726109. 22431615 PMC3325727

[37] Hatori M, Vollmers C, Zarrinpar A, DiTacchio L, Bushong EA, Gill S, Leblanc M, Chaix A, Joens M, Fitzpatrick JAJ, Ellisman MH, Panda S 2012 Time-restricted feeding without reducing caloric intake prevents metabolic diseases in mice fed a high-fat diet. Cell Metab 15:848–860. doi:10.1016/j.cmet.2012.04.019. 22608008 PMC3491655

[38] Ye C, Wang R, Tai Y, Zhang LH, Tang SH, Tang CW 2018 Obesity damages intestinal mucosal barrier and microbiota composition in rat model of severe acute pancreatitis. Gastroenterology 154:S-948. doi:10.1016/S0016-5085(18)33195-0.

[39] Paolella G, Vajro P 2018 Maternal microbiota, prepregnancy weight, and mode of delivery intergenerational transmission of risk for childhood overweight and obesity. JAMA Pediatr 172:320–322. doi:10.1001/jamapediatrics.2017.5686. 29459936

[40] Foley KP, Zlitni S, Denou E, Duggan BM, Chan RW, Stearns JC, Schertzer JD 2018 Long term but not short term exposure to obesity related microbiota promotes host insulin resistance. Nat Commun 9:4681. doi:10.1038/s41467-018-07146-5. 30409977 PMC6224578

[41] Joyce SA, MacSharry J, Casey PG, Kinsella M, Murphy EF, Shanahan F, Hill C, Gahan C 2014 Regulation of host weight gain and lipid metabolism by bacterial bile acid modification in the gut. Proc Natl Acad Sci U S A 111:7421–7426. doi:10.1073/pnas.1323599111. 24799697 PMC4034235

[42] Torres-González LA, Rodríguez-León O, Alvarado-Carrillo V, Escogido LR 2013 Network modeling of gene expression microarray in patients with obesity and relationship with lactobacillus probiotic intake. Proc Nutr Soc 72:E80. doi:10.1017/S0029665113000827.

[43] Naito E, Yoshida Y, Makino K, Kounoshi Y, Kunihiro S, Takahashi R, Matsuzaki T, Miyazaki K, Ishikawa F 2011 Beneficial effect of oral administration of *Lactobacillus casei* strain Shirota on insulin resistance in diet-induced obesity mice. J Appl Microbiol 110:650–657. doi:10.1111/j.1365-2672.2010.04922.x. 21281408

[44] Thaiss CA, Levy M, Korem T, Dohnalova L, Shapiro H, Jaitin DA, David E, Winter DR, Gury-BenAri M, Tatirovsky E, Tuganbaev T, Federici S, Zmora N, Zeevi D, Dori-Bachash M, Pevsner-Fischer M, Kartvelishvily E, Brandis A, Harmelin A, Shibolet O, Halpern Z, Honda K, Amit I, Segal E, Elinav E 2016 Microbiota diurnal rhythmicity programs host transcriptome oscillations. Cell 167:1495.e12–1510.e12. doi:10.1016/j.cell.2016.11.003. 27912059

[45] Karasek M, Winczyk K 2006 Melatonin in humans. J Physiol Pharmacol 57(Suppl 5):19–39. 17218758

[46] Jung MJ, Lee J, Shin NR, Kim MS, Hyun DW, Yun JH, Kim PS, Whon TW, Bae JW 2016 Chronic repression of mTOR complex 2 induces changes in the gut microbiota of diet-induced obese mice. Sci Rep 6:30887. doi:10.1038/srep30887. 27471110 PMC4965768

[47] Galley JD, Bailey M, Dush CK, Schoppe-Sullivan S, Christian LM 2014 Maternal obesity is associated with alterations in the gut microbiome in toddlers. PLoS One 9:e113026. doi:10.1371/journal.pone.0113026. 25409177 PMC4237395

[48] Gunn PJ, Middleton B, Davies SK, Revell VL, Skene DJ 2016 Sex differences in the circadian profiles of melatonin and cortisol in plasma and urine matrices under constant routine conditions. Chronobiol Int 33:39–50. doi:10.3109/07420528.2015.1112396. 26731571 PMC4819823

[49] Yin J, Li YY, Zhu XT, Han H, Ren WK, Chen S, Bin P, Liu G, Huang XG, Fang RJ, Wang B, Wang K, Sun LP, Li TJ, Yin YL 2017 Effects of long-term protein restriction on meat quality, muscle amino acids, and amino acid transporters in pigs. J Agric Food Chem 65:9297–9304. doi:10.1021/acs.jafc.7b02746. 28965404

[50] Talbot CPJ, Plat J, Ritsch A, Mensink RP 2018 Determinants of cholesterol efflux capacity in humans. Prog Lipid Res 69:21–32. doi:10.1016/j.plipres.2017.12.001. 29269048

[51] Pirro M, Ricciuti B, Rader DJ, Catapano AL, Sahebkar A, Banach M 2018 High density lipoprotein cholesterol and cancer: marker or causative? Prog Lipid Res 71:54–69. doi:10.1016/j.plipres.2018.06.001. 29879431

[52] Maraschin FDS, Kulcheski FR, Segatto ALA, Trenz TS, Barrientos-Diaz O, Margis-Pinheiro M, Margis R, Turchetto-Zolet AC 2019 Enzymes of glycerol-3-phosphate pathway in triacylglycerol synthesis in plants: function, biotechnological application and evolution. Prog Lipid Res 73:46–64. doi:10.1016/j.plipres.2018.12.001. 30521822

[53] Yu XH, Zhang DW, Zheng XL, Tang CK 2019 Cholesterol transport system: an integrated cholesterol transport model involved in atherosclerosis. Prog Lipid Res 73:65–91. doi:10.1016/j.plipres.2018.12.002. 30528667

[54] Yin J, Han H, Li Y, Liu Z, Zhao Y, Fang R, Huang X, Zheng J, Ren W, Wu F, Liu G, Wu X, Wang K, Sun L, Li C, Li T, Yin Y 2017 Lysine restriction affects feed intake and amino acid metabolism via gut microbiome in piglets. Cell Physiol Biochem 44:1749–1761. doi:10.1159/000485782. 29216634

[55] Yook JS, Kim KA, Kim M, Cha YS 2017 Black adzuki bean (*Vigna angularis*) Attenuates high-fat diet-induced colon inflammation in mice. J Med Food 20:367–375. doi:10.1089/jmf.2016.3821. 28406732

[56] Yin J, Ren W, Duan J, Wu L, Chen S, Li T, Yin Y, Wu G 2014 Dietary arginine supplementation enhances intestinal expression of SLC7A7 and SLC7A1 and ameliorates growth depression in mycotoxin-challenged pigs. Amino Acids 46:883–892. doi:10.1007/s00726-013-1643-5. 24368521

[57] Yin J, Ren W, Liu G, Duan J, Yang G, Wu L, Li T, Yin Y 2013 Birth oxidative stress and the development of an antioxidant system in newborn piglets. Free Radic Res 47:1027–1035. doi:10.3109/10715762.2013.848277. 24074241

[58] Yin J, Wu MM, Xiao H, Ren WK, Duan JL, Yang G, Li TJ, Yin YL 2014 Development of an antioxidant system after early weaning in piglets. J Anim Sci 92:612–619. doi:10.2527/jas.2013-6986. 24352957

[59] Yin J, Liu M, Ren W, Duan J, Yang G, Zhao Y, Fang R, Chen L, Li T, Yin Y 2015 Effects of dietary supplementation with glutamate and aspartate on diquat-induced oxidative stress in piglets. PLoS One 10:e0122893. doi:10.1371/journal.pone.0122893. 25875335 PMC4398417

[60] Yin J, Li Y, Han H, Zheng J, Wang L, Ren W, Chen S, Wu F, Fang R, Huang X, Li C, Tan B, Xiong X, Zhang Y, Liu G, Yao J, Li T, Yin Y 2017 Effects of lysine deficiency and Lys-Lys dipeptide on cellular apoptosis and amino acids metabolism. Mol Nutr Food Res 61:1600754. doi:10.1002/mnfr.201600754. 28012236

[61] Yin J, Wu M, Duan J, Liu G, Cui Z, Zheng J, Chen S, Ren W, Deng J, Tan X, Al-Dhabi NA, Duraipandiyan V, Liao P, Li T, Yulong Y 2015 Pyrrolidine dithiocarbamate inhibits NF-kappaB activation and upregulates the expression of Gpx1, Gpx4, occludin, and ZO-1 in DSS-induced colitis. Appl Biochem Biotechnol 177:1716–1728. doi:10.1007/s12010-015-1848-z. 26386585

[62] Kashinskaya EN, Simonov EP, Kabilov MR, Izvekova GI, Andree KB, Solovyev MM 2018 Diet and other environmental factors shape the bacterial communities of fish gut in an eutrophic lake. J Appl Microbiol 125:1626–1641. doi:10.1111/jam.14064. 30091826

[63] Castillo-Lopez E, Moats J, Aluthge ND, Ramirez HAR, Christensen DA, Mutsvangwa T, Penner GB, Fernando SC 2018 Effect of partially replacing a barley-based concentrate with flaxseed-based products on the rumen bacterial population of lactating Holstein dairy cows. J Appl Microbiol 124:42–57. doi:10.1111/jam.13630. 29112793

[64] Aßhauer KP, Wemheuer B, Daniel R, Meinicke P 2015 Tax4Fun: predicting functional profiles from metagenomic 16S rRNA data. Bioinformatics 31:2882–2884. doi:10.1093/bioinformatics/btv287. 25957349 PMC4547618

[65] Hiragaki S, Baba K, Coulson E, Kunst S, Spessert R, Tosini G 2014 Melatonin signaling modulates clock genes expression in the mouse retina. PLoS One 9:e106819. doi:10.1371/journal.pone.0106819. 25203735 PMC4159264

