# Supplementary figures and images for: Administration of Exogenous Melatonin Improves the Diurnal Rhythms of the Gut Microbiota in Mice Fed a High-Fat Diet
Source: mSystems. 2020 May 19;5(3):e00002-20. doi: 10.1128/mSystems.00002-20 (PMC7253360; doi:10.1128/mSystems.00002-20)

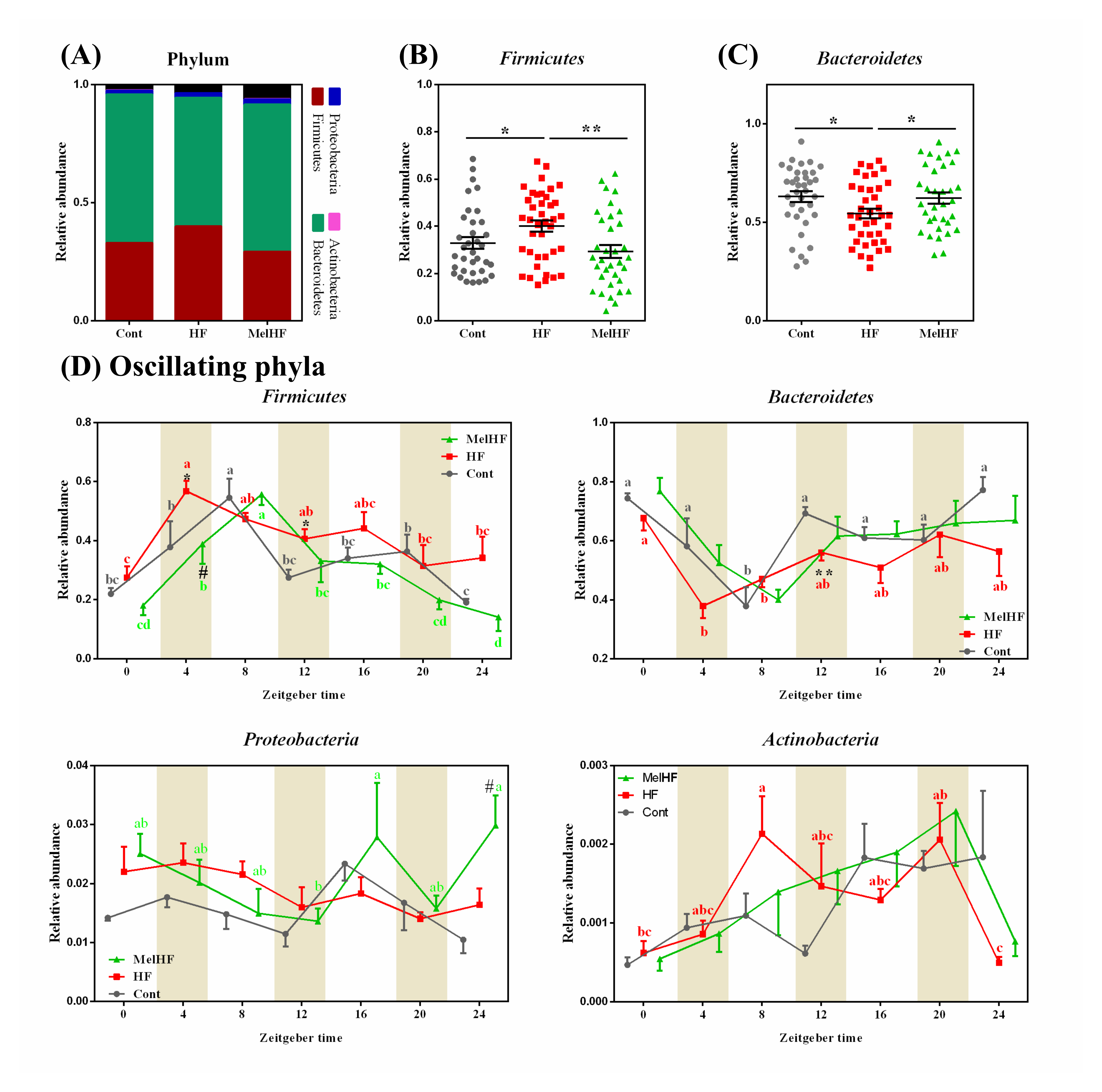

Supplement: FIG S1 [file mSystems.00002-20-sf001.tif]

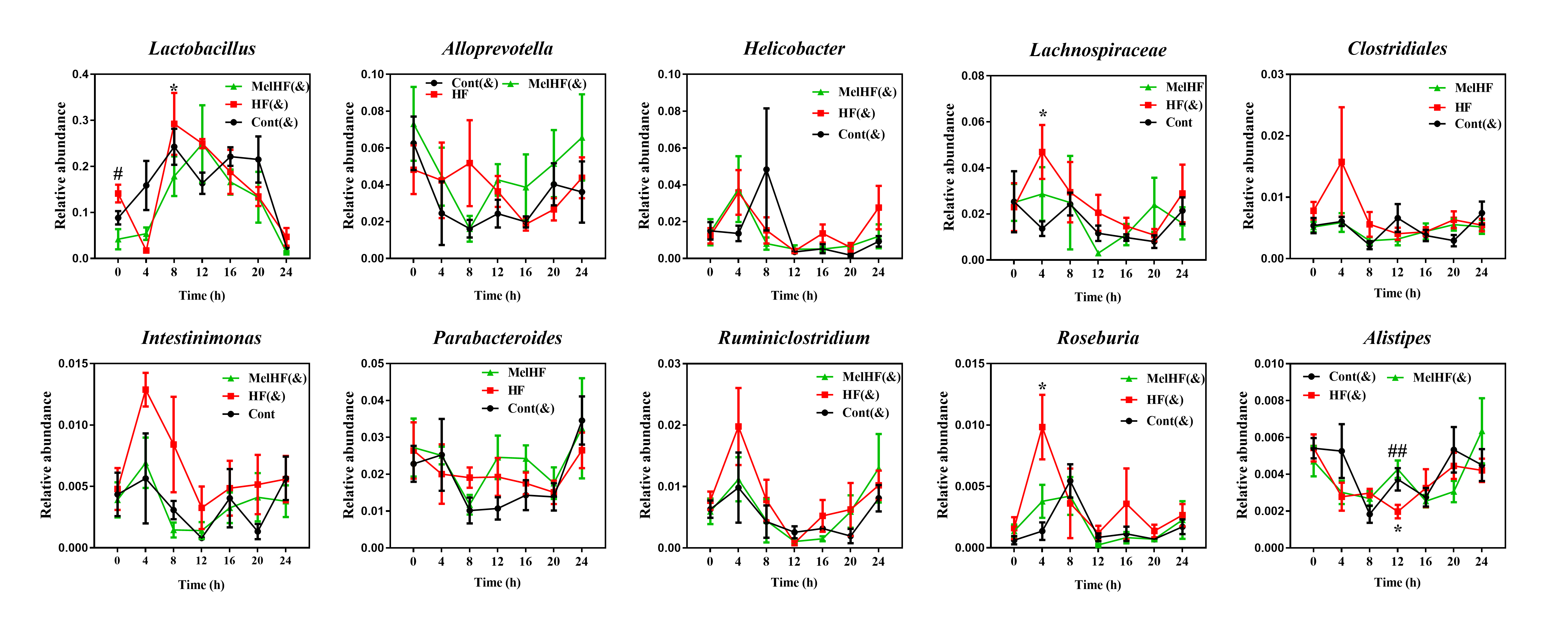

Supplement: FIG S2 [file mSystems.00002-20-sf002.tif]

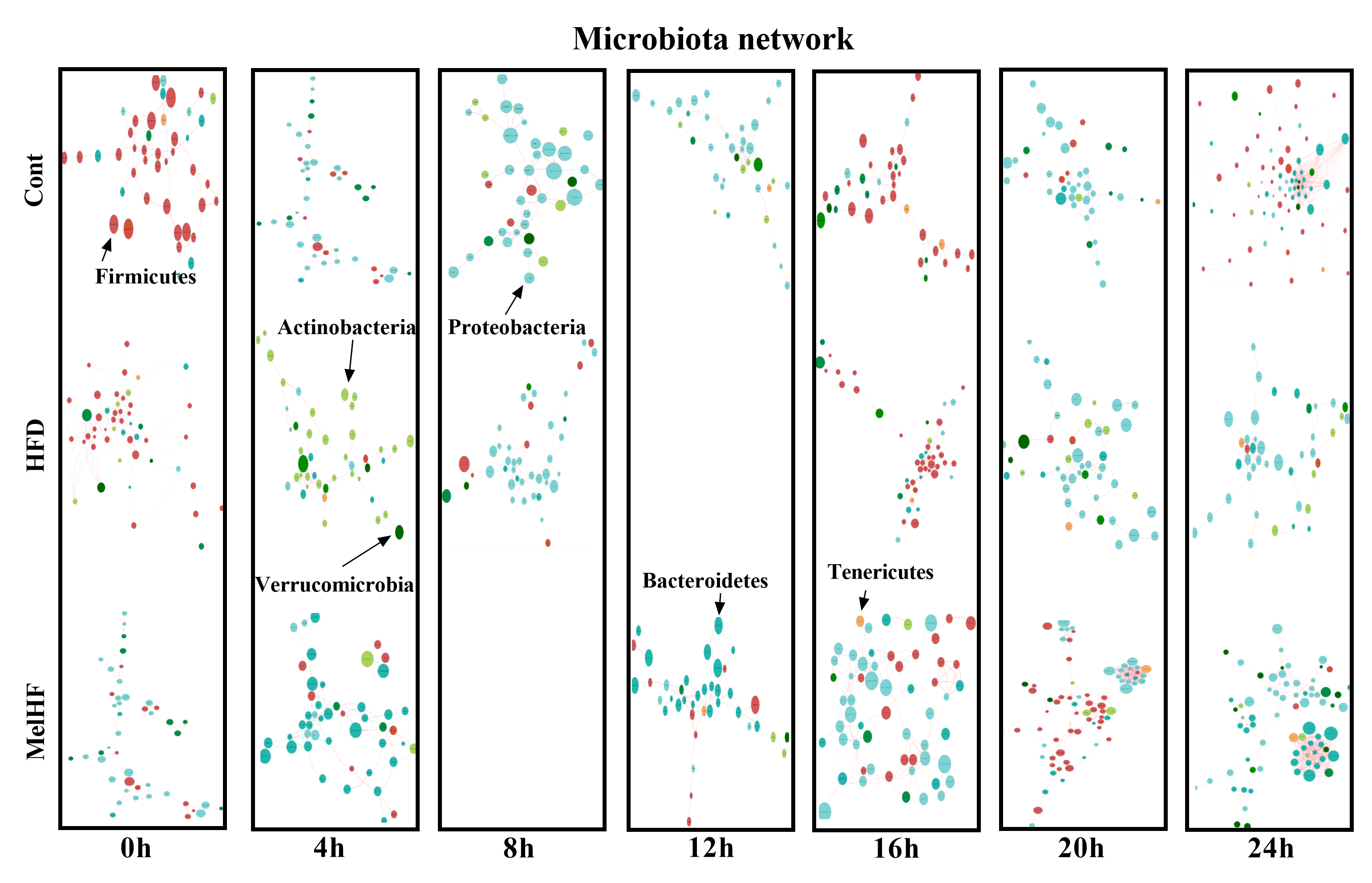

Supplement: FIG S3 [file mSystems.00002-20-sf003.tif]

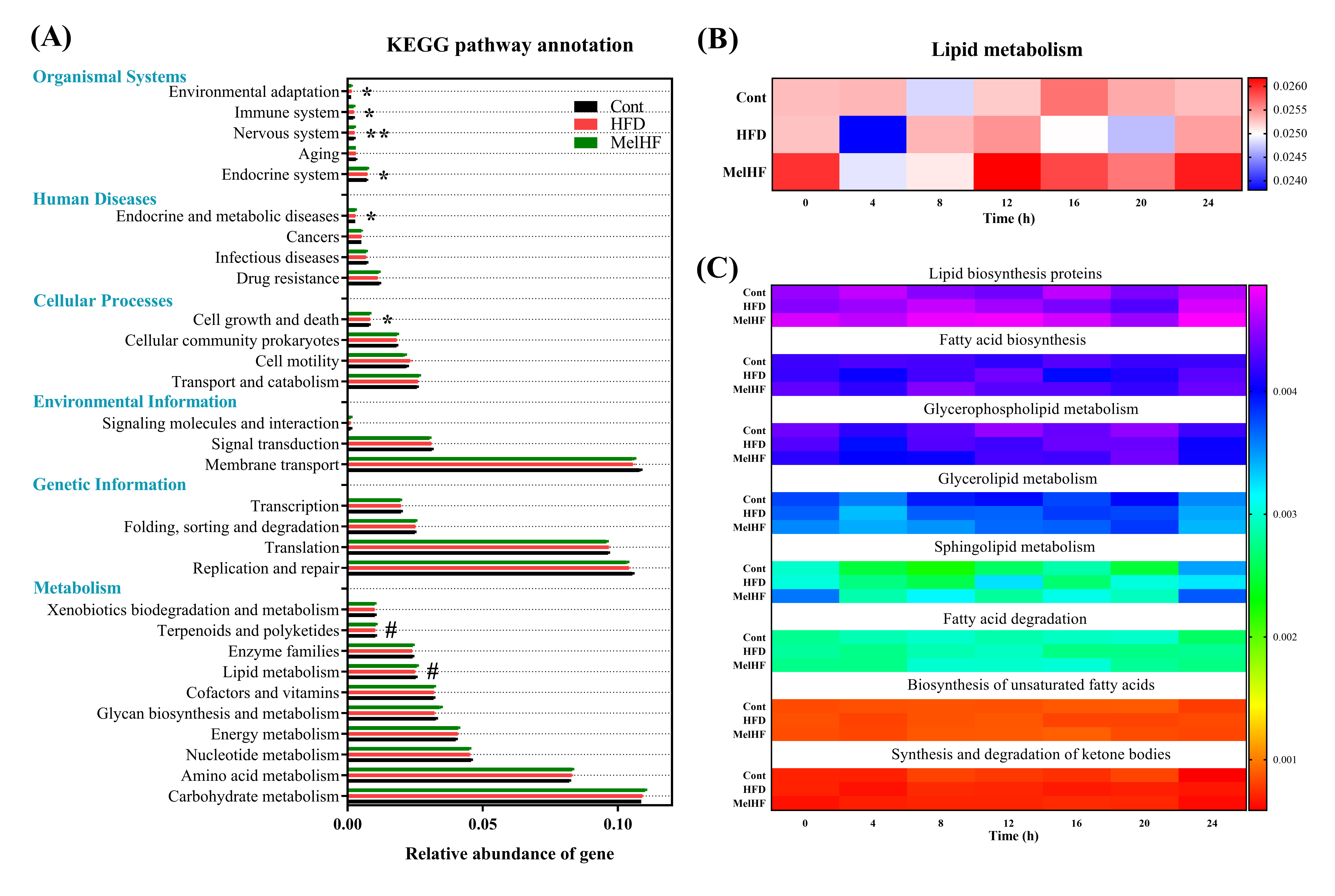

Supplement: FIG S4 [file mSystems.00002-20-sf004.tif]
