# Supplementary material for: Administration of Exogenous Melatonin Improves the Diurnal Rhythms of the Gut Microbiota in Mice Fed a High-Fat Diet
Source: mSystems. 2020 May 19;5(3):e00002-20. doi: 10.1128/mSystems.00002-20 (PMC7253360; doi:10.1128/mSystems.00002-20)
Supplement: TABLE S1 [file mSystems.00002-20-st001.docx]

**Supplementary Table 1.** Primers used in this study.

*Clock*

forward: AGACGGCGAGAACTTGGCATTG

reverse: TTCCTTGAGACTCACTGTGTTGATACG

*Per1*

forward: CTCTTCTGGCAATGGCAAGGACTC

reverse: TCAGGAGGCTGTAGGCAATGGAG

*Per2*

forward: GCTGCGGATGCTCGTGGAATC

reverse: GGTTGTGCTCTGCCTCTGTCATC)

*Cry1*

forward: GCTGGCGTGGAAGTCATCGTG

reverse: GCATCTCCAGTGGCTCCATCTTG)

*Cyr2*

forward: TGGACAAGCACTTGGAACGGAAG

reverse: GTAGAAGAGGCGGCAGGAGAGG)

*β-actin*

forward: GTCCACCTTCCAGCAGATGT

reverse: GAAAGGGTGTAAAACGCAGC)
